# Preheated and Injected Bulk-Fill Resin Composites: A Micro-CT Analysis of Internal Voids and Marginal Adaptation in Class II Restorations

**DOI:** 10.3390/ma18020327

**Published:** 2025-01-13

**Authors:** Vanessa Alves de Sá, Hélio Radke Bittencourt, Luiz Henrique Burnett Júnior, Ana Maria Spohr

**Affiliations:** 1 Department of Restorative Dentistry, School of Dentistry, Pontifical Catholic University of Rio Grande do Sul, Porto Alegre 90619-900, Brazil; vanessaalvessa@hotmail.com (V.A.d.S.); heliorb@pucrs.br (H.R.B.); burnett@pucrs.br (L.H.B.J.); 2 Department of Statistics, Polytechnic School, Pontifical Catholic University of Rio Grande do Sul, Porto Alegre 90690-900, Brazil

**Keywords:** resin composites, temperature, dental marginal adaptation

## Abstract

The aim of this study was to evaluate, in vitro, the void formation and marginal adaptation in Class II cavities restored with preheated and injected bulk-fill resin composites. Eighty third molars received Class II cavities on their mesial and distal surfaces and were randomly distributed into eight groups (n = 10) according to material (Filtek Universal—control, incremental technique; Filtek One Bulk-Fill; Admira Fusion X-tra Bulk-Fill; VisCalor Bulk-Fill) and the temperature of the material (24 °C or 68 °C). The restored teeth were scanned using a SkyScan 1173 microtomograph. The percentage of internal voids (%IV) was analyzed using CTan software (version 1.23.02) and the percentages of continuous margins (%CM) in enamel and dentin were analyzed using Dataviewer software (version 1.5.6.2). The data of %IV and %CM were subjected to two-way ANOVA on ranks, followed by Tukey’s test (α = 0.05). At 24 °C, Filtek Universal had a greater %IV (1.89%) (*p* < 0.05), which did not differ significantly from that of Admira Fusion X-tra Bulk-Fill (0.29%) (*p* > 0.05). Filtek One Bulk-Fill (0.07%) and VisCalor Bulk-Fill (0.07%) had lower %IV (*p* < 0.05). Preheating resulted in a significantly lower %IV for Admira Fusion X-tra Bulk-Fill (*p* < 0.05). Temperature did not significantly influence marginal adaptation (*p* > 0.05). VisCalor Bulk-Fill achieved significantly higher %CM in dentin (98%) at 24 °C (*p* < 0.05). It was concluded that bulk-fill-injected resin composites tend to have fewer internal voids than conventional resin composites using the incremental technique, and the injection of the resin composite into the cavity seems to be more important for marginal adaptation than the preheating procedure.

## 1. Introduction

Bulk-fill resin composites were developed to simplify the restorative technique. These materials can be placed and adequately polymerized in a single layer of 4–5 mm thickness [1], often allowing a single-increment application and reducing the chance of void formation [2,3]. These materials can be classified into low-viscosity and high-viscosity material [4]. However, the application of high-viscosity resin composites into narrow and deep cavities is challenging, leading to poor adaptation to walls and angles [5,6]. To increase the flowability of high-viscosity resin composites, different techniques, such as sonic vibration and preheating, have been proposed [7].

The preheating of conventional resin composites to 54 °C or 68 °C has been used to temporarily reduce the viscosity of the material during application [8,9]. The effect of preheating on viscosity depends on the resin composite’s composition and commercial brand [10]. The viscosity of conventional resin composites ranged from 6.75 to 19.14 kPa·s at 23 °C, and preheating to 54 °C led to a reduction of 30–82% in viscosity [11]. This technique is considered a simple, safe, and practical approach [12]. Additionally, it improves the adaptation of the material to cavity walls [12,13,14] without compromising the mechanical properties of the material [12,15,16].

Currently, various bulk-fill resin composites are available, but not all of them are recommended for preheating by the manufacturer. Recently, a thermoviscous bulk-fill resin composite, called VisCalor, was introduced on the market. This material is specifically designed for viscosity modulation by heating. The manufacturer claims that the material utilizes a novel thermoviscous technology linked to the treatment of the filler surface and the incorporation of a coordinated composite matrix. This material demonstrated a significantly greater reduction in viscosity than conventional resin composites. The viscosity of VisCalor decreased from 11.59 kPa·s at 23 °C to 2.06 kPa·s at 54º C [11]. In addition, this resin composite exhibited better internal adaptation [13] and a lower percentage of internal voids (2.99%) compared to a conventional resin composite (5.52%) and a bulk-fill resin composite (3.19%) [17], as well as better external adaptation [18].

Given the variability of preheating and the different bulk-fill resin composites, further studies comparing the formation of internal voids as well as the marginal adaptation of these materials in Class II cavities are lacking. Therefore, the aim of this study was to evaluate, in vitro, the formation of internal voids and marginal adaptation in Class II restorations using bulk-fill resin composites preheated and at room temperature with the injectable technique. This study was based on the hypotheses that (i) the resin composite and (ii) the temperature of the resin composite significantly influence the formation of internal voids and marginal adaptation.

## 2. Materials and Methods

### 2.1. Ethical Aspects

This research was approved by a local ethics committee (CAAE 74950223.9.0000.5336) on 21 July 2023. Eighty human third molars free of caries, restorations, hypoplastic defects, or fractures were obtained with the signing of the consent form for tooth donation. The teeth were cleaned and stored in distilled water at 4 °C.

### 2.2. Cavity Preparation

Standardized Class II box-shaped preparations were made on the mesial and distal surfaces of each tooth. The preparations were carried out at high speed with cooling using a cylinder-shaped diamond rotary instrument with a flat end and rounded edges (#3100, KG Sorensen, São Paulo, SP, Brazil) positioned along the long axis of the tooth. The dimensions of the cavity were 5 mm buccolingually and 3 mm mesiodistally on the gingival wall. The gingival margin was positioned 0.5 mm above the enamel–cementum junction (enamel) or 0.5 mm below the enamel–cementum junction (dentin). The gingival margin in the enamel and dentin alternated between the mesial and distal surfaces. The buccal and lingual walls were parallel and perpendicular to the gingival wall. The axial wall was perpendicular to the gingival wall, and the internal angles were rounded. The diamond burs were changed every five cavity preparations. The cavities were prepared by a single operator.

### 2.3. Restorative Materials and Group Assignment

The teeth were randomly distributed into eight groups (n = 10) according to the resin composition and material temperature (Table 1). Group 1 was considered the control. Information on the materials used in this study is presented in Table 2.

### 2.4. Restorative Technique

The tooth with the cavity preparations was inserted into a dental model (MOM, Marília, SP, Brazil) in the lower right first molar position and stabilized with wax (Wax Pink 7, Lysanda, São Paulo, SP, Brazil). Plastic teeth corresponding to the lower right second premolar and lower right second molar were placed in the dental model to simulate the technical challenges of proximal restorations. The dental model was inserted into a dental phantom head (MOM, Marília, SP, Brazil) to simulate clinical conditions. All restorations were performed by a single right-handed operator seated on a dental stool and working between the 9:00 and 12:00 positions. The height of the dental phantom mouth was adjusted such that it was near the level of the operator’s elbow. The ambient temperature was maintained at 24 °C (±1 °C).

For the restorative procedure, selective enamel etching was performed with 35% phosphoric acid (Ultra-Etch IndiSpense, Ultradent, Indaiatuba, SP, Brazil) for 30 s, followed by rinsing and drying with an air blast. A layer of Single Bond Universal Plus adhesive (3M, St. Paul, MN, USA) was applied to both enamel and dentin using a microbrush on all surfaces of the preparation. The adhesive was rubbed into the dentin for 20 s, followed by a light air blast for 5 s. The adhesive was light-cured for 10 s with a curing unit (GRAND VALO, Ultradent, South Jordan, UT, USA) with an intensity of approximately 1000 mW/cm^2^. A Palodent 360 matrix band (Dentsply Sirona, New York, NY, USA) was placed around the tooth. A plastic wedge was placed between the prepared tooth and the adjacent teeth to prevent resin composite from overflowing beyond the gingival margin. The restorations were carried out as described in Table 1.

Group 1 corresponded to the control group, and the hand incremental technique was applied. Each 2 mm thick increment of Filtek Universal was measured by using a periodontal probe. The first increment was taken with a spatula (Ball Burnisher, Quinelato, Rio Claro, SP, Brazil), placed into the preparation in a horizontal layer and pushed toward the gingival margin with the spatula. The other increments were applied obliquely until the cavity was filled using another spatula (Aplicca Twist, Quinelato, Rio Claro, SP, Brazil). Each increment was light-cured for 20 s.

In Groups 3, 5, and 7, the resin composites were directly injected into the cavity from the capsule at room temperature (24 °C). The tip of the capsule was placed in contact with the gingival wall, and the material was extruded into the cavity in a bulk increment, followed by light curing for 20 s.

In Groups 2, 4, 6, and 8, the resin composites were preheated. The HotRon heater (Biotron, Santa Rita do Sapucaí, MG, Brazil) was kept on for 35 min to reach a heating temperature of 68 °C according to the manufacturer’s instructions. After this time, the capsules were kept in the heater for 3 min. Then, the resin composites were directly injected into the cavity from the capsule as described for Groups 3, 5, and 7 and light-cured for 20 s.

The same device for extruding the resin composites from the capsules was used (3M, St. Paul, MN, USA), which was compatible with all the capsules. After the restorative procedure, the teeth were removed from the dental model and stored in distilled water.

### 2.5. Scanning Procedures

The restored teeth were scanned at high resolution with a SkyScan 1173 computerized X-ray microtomograph (Bruker, Kontich, Belgium) using SkyScan 1173 software. The scanning parameters were 100 kVp, 80 mA, 1.0 mm Al filter, 10 μm pixel size, and 0.25° rotation step. Each tooth was scanned 270° within an integration time of 2 min. To minimize ring artifact noise, detector calibration was performed before each scan. The average scan time for each sample was approximately 50 min.

### 2.6. Void Analysis

The two restorations (mesial and distal) were evaluated separately and randomly. The region of interest (RI) was determined after reconstruction using CTAn visualization software (Bruker, Kontich, Belgium), version 1.23.02, through 3D microarchitecture analysis for each sample. The RI included the entire restoration within the tooth. Unwanted voxels were adjusted for brightness and opacity before the voids were visualized and calculated. The internal voids in the 3D volume were calculated using the original grayscale images processed with a Gaussian filter to reduce noise. The enamel and dentin were subtracted from the restorations, allowing for visualization and evaluation of only the internal voids within the RI using an automatic segmentation threshold in the CTAn image analysis software, version 1.23.02. A global threshold was used to process grayscale level ranges to obtain an image consisting only of black and white pixels. The volumes of internal voids relative to the total restoration volume were calculated as percentages (%).

### 2.7. Marginal Adaptation Analysis

The Dataviewer software (Bruker, Kontich, Belgium), version 1.5.6.2, performed the reconstruction of the samples, allowing the images to be viewed in three orthogonal planes (vertical, horizontal, and perpendicular depth) slice by slice, enabling three-dimensional (3D) analysis. The RI was selected in the vertical direction (long axis of the tooth) and in the mesial or distal region at the outermost portion of the restoration, to visualize the external adaptation of the resin composite at the gingival margin. Using the software’s ruler, the total length of the gingival margin of the restoration (buccal-lingual direction) was measured in millimeters (mm), as was the length of any gap when present in the enamel and dentin separately. The percentage (%) of continuous margins was calculated for each restoration.

### 2.8. Sample Size Calculation and Statistical Analysis

The convenience sample was comprised of ten teeth per group (n = 10), which was based on the specialized literature [13,14,19]. This study includes two factors (composite resin × temperature). The data on internal voids and the percentage of continuous margins did not follow a normal distribution according to the Shapiro-Wilk test, and there was no homogeneity according to the Levene’s test. Therefore, a nonparametric test should be chosen. However, because there is no direct nonparametric test equivalent to a two-way ANOVA, we followed Conover’s suggestion [20]. The data were transformed into ranks, and a two-way ANOVA was performed on these ranks, followed by the Tukey test. The significance level was set at 5% (*p* = 0.05).

## 3. Results

### 3.1. Percentage (%) of Internal Voids

The factors resin composite (*p* = 0.0001) and temperature (*p* = 0.001) and the interaction between these factors (*p* = 0.0001) were significant.

At 24 °C, Filtek Universal resulted in a greater percentage of internal voids (*p* < 0.05), which was not significantly different from that of Admira Fusion X-tra Bulk-Fill (*p* > 0.05). Filtek One Bulk-Fill and VisCalor Bulk-Fill had lower percentages of internal voids (*p* < 0.05). Preheating significantly decreased the percentages of internal voids for Filtek Universal and Admira Fusion X-tra Bulk-Fill (*p* < 0.05) (Table 3).

**Figure 1:**
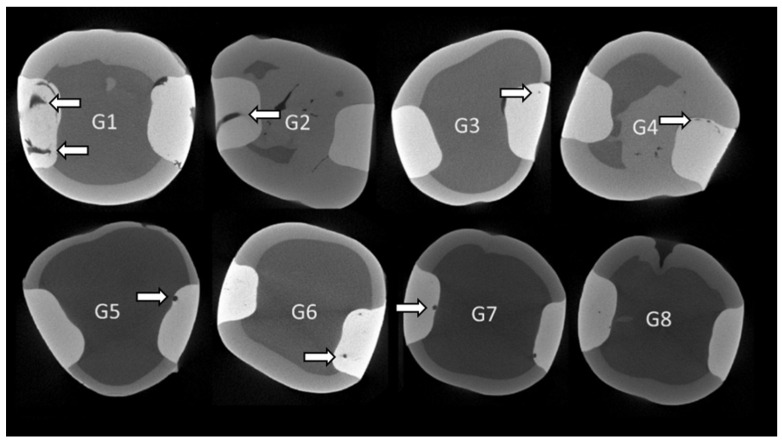
Micro-CT images of void formation. The black areas inside the resin composite applied in the Class II cavities correspond to voids (arrows) in the material. G1—Filtek Universal (incremental technique) at room temperature; G2—Filtek Universal (injected) preheated at 68 °C; G3—Filtek One Bulk-Fill (injected) at room temperature; G4—Filtek One Bulk-Fill (injected) preheated at 68 °C; G5—Admira Fusion X-tra Bulk-Fill (injected) at room temperature; G6—Admira Fusion X-Tra Bulk-Fill (injected) preheated at 68 °C; G7—VisCalor Bulk-Fill (injected) at room temperature; G8—VisCalor Bulk-Fill (injected) preheated at 68 °C.

Figure 1 shows the internal voids of the experimental groups.

### 3.2. Percentage (%) of Continuous Margins in Enamel

The resin composite factor was significant (*p* = 0.0001), whereas the temperature factor (*p* = 0.216) and the interaction between the factors (*p* = 0.557) were not significant for the enamel margin.

At 24 °C and 68 °C, VisCalor Bulk-Fill presented the highest percentage of continuous margins (*p* < 0.05), which was not significantly different from those of Filtek One Bulk-Fill and Admira Fusion X-tra Bulk-Fill (*p* > 0.05). Filtek Universal had the lowest percentage of continuous margins (*p* < 0.05), which was not significantly different from that of Filtek One Bulk-Fill (*p* > 0.05) (Table 4).

### 3.3. Percentage (%) of Continuous Margins in Dentin

The resin composite factor was significant (*p* = 0.0001), whereas the temperature factor (*p* = 0.097) and the interaction between the factors (*p* = 0.469) were not significant for the dentin margin.

At 24 °C, VisCalor Bulk-Fill achieved the highest percentage of continuous margins, differing significantly from the other groups (*p* < 0.05). Filtek Universal had the lowest percentage of continuous margins (*p* < 0.05), which was not significantly different from that of Filtek One Bulk-Fill (*p* > 0.05). At 68 °C, VisCalor Bulk-Fill achieved the highest percentage of continuous margins (*p* < 0.05), which did not differ significantly from that of Admira Fusion X-tra Bulk-Fill (*p* > 0.05). Filtek Universal had the lowest percentage of continuous margins (*p* < 0.05), which was not significantly different from that of Filtek One Bulk-Fill (*p* > 0.05) (Table 5).

**Figure 2:**
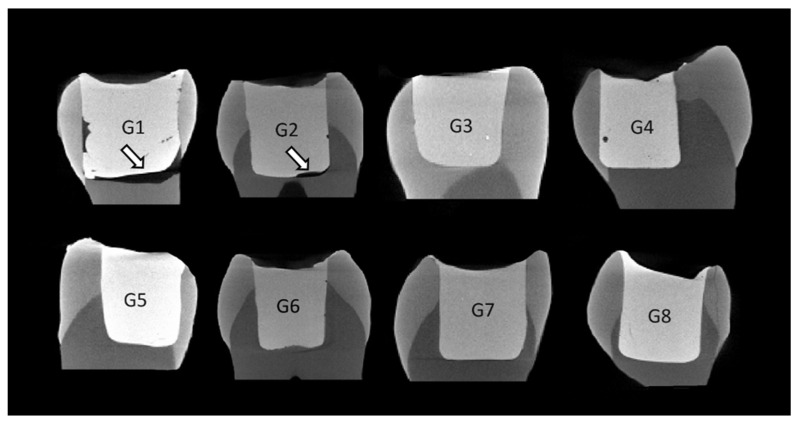
Micro-CT images representing the marginal adaptation analysis in the cervical margin at dentin for the different groups. The arrows indicate marginal gaps. G1—Filtek Universal (incremental technique) at room temperature; G2—Filtek Universal (injected) preheated at 68 °C; G3—Filtek One Bulk-Fill (injected) at room temperature; G4—Filtek One Bulk-Fill (injected) preheated at 68 °C; G5—Admira Fusion X-tra Bulk-Fill (injected) at room temperature; G6—Admira Fusion X-Tra Bulk-Fill (injected) preheated at 68 °C; G7—VisCalor Bulk-Fill (injected) at room temperature; G8—VisCalor Bulk-Fill (injected) preheated at 68 °C.

Figure 2 shows the micro-CT images of samples from the different groups, highlighting the marginal adaptation at the cervical level.

## 4. Discussion

In light of the trend towards simplifying the restorative technique, the present study tested two bulk-fill resin composites (Filtek One Bulk-Fill and Admira Fusion X-tra Bulk-Fill) and a thermoviscous bulk-fill resin composite (Viscalor Bulk-Fill). These resin composites use the injection technique, which is a current trend, and the effect of preheating on internal voids and marginal adaptation was evaluated. The control group consisted of a conventional resin composite (Filtek Universal) applied using the incremental technique at room temperature.

The first outcome evaluated was the percentage of internal voids, and significant differences were found among the resin composites. Thus, the hypothesis that the resin composite influences the formation of internal voids was accepted.

Filtek Universal (Group 1—control) is a conventional resin composite provided in a tube when preheating is not applied. This material requires an incremental technique to ensure adequate polymerization of the entire application, especially in the deeper regions of the cavity [21,22]. However, this technique favored greater void formation due to air entrapment between the successive layers of the resin composite, corroborating the findings of other studies [2,3,23]. Conversely, bulk-fill resin composites have a greater depth of cure, allowing for a single-increment application and reducing the chance of creating internal voids due to air entrapment between layers [2,3].

Comparing the three bulk-fill resin composites at room temperature, the Admira Fusion X-tra Bulk-Fill had a greater percentage of internal voids (0.29%) and did not differ significantly from Filtek Universal (1.89%). Among the tested bulk-fill resin composites, the Admira Fusion X-tra Bulk-Fill composite has the highest filler content (84.0 wt%). Generally, a higher filler content tends to increase the viscosity of the material [24]. Therefore, when extruded from the capsule into the cavity, it is assumed that the higher viscosity of this resin composite contributed to the greater formation of internal voids. In addition, void formation may also result from the resin composite manufacturing process [25], which could also explain the differing percentages of internal voids among the bulk-fill resin composites.

Preheating favored decreases in the percentages of internal voids for Filtek Universal and Admira Fusion X-tra Bulk-Fill, whereas this procedure did not produce significant differences for Filtek One Bulk-Fill and VisCalor Bulk-Fill. Thus, the hypothesis that preheating significantly influences the formation of internal voids was partially accepted.

In Group 2, Filtek Universal is provided in a capsule to preheat the material. Although the manufacturer recommends application in increments of 2 mm, this resin composite was applied in a single increment. The insertion in a single increment was performed for the following reasons: (a) measuring increments of 2 mm thickness using a preheated injectable resin composite was technically difficult; and (b) the aim was to eliminate the air entrapment between layers to observe the internal voids resulting from the injection process itself associated with preheating. Importantly, this procedure was performed for research purposes and is not clinically recommended. The combination of single-increment injection and preheating contributed to a significant decrease in void formation for Filtek Universal, from 1.89% to 0.26%, indicating that void formation in preheated Filtek Universal is not related to air entrapment between layers but rather to internal voids within the material itself. When the resin composites are delivered in capsules, the movement of the plunger and the exit of the material through the nozzle can also introduce air bubbles and internal voids [26,27]. This may explain why a void-free technique was not achieved in this study even when a single increment was used. Furthermore, according to the manufacturer of VisCalor Bulk-Fill, the capsule of this resin composite has a narrow and long tip that allows for bubble-free restoration, which was not confirmed by the present study, as internal voids were observed.

A significant decrease in the percentage of internal voids also occurred for preheated Admira Fusion X-tra Bulk-Fill. Because this resin composite had the highest percentage of internal voids at room temperature (0.29%) among the tested bulk-fill materials, the effect of preheating was more evident, decreasing void formation to 0.03% by reducing the viscosity of the material [17].

For Filtek One Bulk-Fill and VisCalor Bulk-Fill, preheating did not significantly influence the percentage of internal voids. These findings do not corroborate the study by Demirel et al., which revealed a significant decrease in the percentage of internal voids when Filtek One Bulk-Fill and VisCalor Bulk-Fill were applied using the preheating technique [17]. The different results obtained in these studies are probably due to differences in research methodologies, light-curing times, or even technical and operator variability. Furthermore, void formation is a multifactorial phenomenon that is influenced by the polymerization of the material, the resin matrix composition, the polymer network architecture, and its heterogeneity [28].

It is important to emphasize that internal voids in a restoration are undesirable porosities that can negatively affect the mechanical properties of the resin composite [14]. These internal voids can affect restorations, particularly under fatigue loading, as they may act as stress risers and lead to fractures and clinical failures in restorations [29]. In general, the percentage of internal voids observed in the present study can be considered low for the injected bulk-fill resin composites. Although a statistically significant difference was observed in the percentage of internal voids among the injected bulk-fill resin composites, it is difficult to determine whether these small differences could clinically influence the longevity of the restorations.

With respect to the percentages of continuous margins in enamel and dentin, there were significant differences among the resin composites. Thus, the hypothesis that the resin composite significantly influences marginal adaptation was accepted.

The incremental technique using Filtek Universal resulted in lower percentages of continuous margins both at the enamel (86.4%) and dentin (75.1%) margins at room temperature. One of the reasons for the gaps may be the stickiness of the resin composite on the metal instrument, which is defined as “the adhesion force between two contracted surfaces” [30,31]. When the material is pressed by a metal instrument toward the gingival margin, monomers from the uncured resin composite can stick to the metal instrument by weak dipole (van der Waals) interactions [11]. If bonding to the metal instrument is stronger than bonding to the adhesive applied on the gingival margin, the material bonded to the instrument may be pulled back when the instrument is removed, leading to the formation of a gap [30]. Once light-cured, the defect is incorporated into the restoration.

Filtek One Bulk-Fill was the only resin composite that did not differ significantly from Filtek Universal at room temperature for both the enamel and dentin margins. Although there was no significant difference, there was a trend toward greater percentages of continuous margins for Filtek One Bulk-Fill (93.7% in enamel and 87.2% in dentin), which was injected into the cavity, than for the incremental technique used for Filtek Universal (86.4% in enamel and 75.1% in dentin) at room temperature. When preheated, Filtek One Bulk-Fill also did not differ significantly from Filtek Universal at either margin. In this case, both resin composites were injected from the capsule in a single increment into the cavity. These resin composites have similar monomers and filler compositions, as well as the same filler percentages. In addition, the tips of the capsules have the same diameter (2 mm). These similarities likely contributed to similar rheological properties when the materials were injected into the cavities.

Both at room temperature and when preheated, Filtek One Bulk-Fill, Admira Fusion X-tra Bulk-Fill, and VisCalor Bulk-Fill presented percentages of continuous margins that did not differ significantly in the enamel margin. However, a trend was observed for a higher percentage of continuous margins for VisCalor Bulk-Fill. This resin composite achieved 100% continuous margins in the enamel margin, which is the desired outcome for clinical situations. The same trend of a greater percentage of continuous margins for VisCalor Bulk-Fill was observed in the dentin margin. The better results for VisCalor can be related to the rheological properties of the material, associated with the smallest nozzle tip diameter (1 mm) compared with the other resin composites (2 mm). It is known that resin composites exhibit non-Newtonian, shear thinning behavior (i.e., the viscosity decreases as the shear rate increases) [32]. When extruding the VisCalor resin composite through a nozzle with a smaller diameter, we hypothesize that the material was subjected to a higher shear rate, resulting in lower viscosity and better adaptation to the cavity.

In the present study, preheating did not significantly improve the marginal adaptation of any resin composite tested. Thus, the hypothesis that temperature influences marginal adaptation was rejected. The manufacturer recommends preheating Filtek Universal (capsules) and VisCalor Bulk-Fill, whereas Filtek One Bulk-Fill and Admira Fusion X-tra Bulk-Fill do not have a preheating recommendation. Preheating reduces the internal friction between the monomers by increasing their molecular vibration, which forces the monomers of the matrix to be further separated. This allows the monomers to slide past each other more easily, increasing the mobility of molecular chains and resulting in a decrease in viscosity [33]. According to the manufacturer, VisCalor Bulk-Fill is the first material to use thermoviscous technology. However, this thermoviscous characteristic was not relevant for better marginal adaptation in the present study. With respect to Filtek One Bulk-Fill, Andrade et al. reported that preheating substantially increased the stickiness of this resin composite and impaired complete adaptation to the angles of the cavity [18]. Thus, better marginal adaptation cannot be expected for all preheated resin composites [18].

The injection of the resin composite inside the cavity seems to be the most important factor influencing marginal adaptation, regardless of the temperature of the material. When the resin composite is injected using an extrusion device, it spreads along the cavity walls and angles through the force created by the plunger inside the capsule. Injection alone has been reported to improve the adaptation of resin composites to walls, decreasing the number of interfacial gaps [34,35,36].

The diameter of the nozzle tip influences the adaptation of the resin composite inside the cavity. If the diameter of the nozzle tip is too large and cannot reach the bottom of the cavity, there is a tendency toward air entrapment and a larger void surface area [26,37]. The nozzle tip diameter of VisCalor Bulk-Fill is 1 mm, while the nozzle tip diameters of the other resin composites are 2 mm. Thus, for all the resin composites, it was possible to initiate material extrusion at the deepest part of the cavity, allowing direct contact with the gingival wall when these materials were injected. Additionally, during material injection, care was taken to avoid separation of the capsule nozzle from the extruding material mass to prevent porosity formation [19].

It is important to emphasize that the formation of marginal gaps depends on interactions among multiple factors. Some factors are related to the resin composite, and others are related to the cavity and the restorative procedure [38,39,40], such as the adhesive system used [41] and the magnitude of the shrinkage stresses generated during the placement and photopolymerization of the resin composite [42].

The clinical relevance of the present study is that the resin composite preheating technique is not essential for better marginal adaptation in Class II cavities for the resin composite tested. In fact, the injection of the resin composite into the cavity appears to be more important for marginal adaptation than the preheating procedure. This is an important finding, as preheating adds an extra clinical step to the restorative procedure. Additionally, the present study showed that none of the resin composites are free of porosities, even when using the injectable technique and preheating.

The limitations of the present study are related to the investigation of only one group of resin composites, given the variety of different commercial brands. Therefore, the findings of this study should not be extrapolated to other resin composites. Although a dental mannequin positioned in a head simulator was used, it is difficult to fully reproduce the clinical conditions. Thus, the results obtained in this study should be interpreted with caution in relation to clinical reality.

## 5. Conclusions

Despite the limitations of this in vitro study, the following conclusions can be drawn for the evaluated resin composites:

- Bulk-fill-injected resin composites tended to have fewer internal voids compared with a conventional resin composite applied using the incremental technique.
- None of the resin composites were free of porosities, and preheating did not contribute to a reduction in internal void formation for the resin composites, except for Admira Fusion X-tra Bulk-Fill.
- Compared with a conventional resin composite, Admira Fusion X-tra Bulk-Fill and VisCalor Bulk-Fill-injected resin composites favored better material adaptation at the cervical margin in Class II cavities.
- Preheating of the resin composites was not relevant for achieving better marginal adaptation at the cervical margin in Class II cavities.

## Figures and Tables

**Table 1:** Study groups.

Groups (n = 10)	Resin Composite	Temperature
1 (control)	Filtek Universal (incremental technique)	Room temperature (24 °C)
2	Filtek Universal (injected)	Preheated (68 °C)
3	Filtek One Bulk-Fill (injected)	Room temperature (24 °C)
4	Filtek One Bulk-Fill (injected)	Preheated (68 °C)
5	Admira Fusion X-tra Bulk-Fill (injected)	Room temperature (24 °C)
6	Admira Fusion X-Tra Bulk-Fill (injected)	Preheated (68 °C)
7	VisCalor Bulk-Fill (injected)	Room temperature (24 °C)
8	VisCalor Bulk-Fill (injected)	Preheated (68 °C)

**Table 2:** Description of the materials used in this study.

Material	Composition	Indication	Batch	Manufacturer
Filtek Universal Tube 4 g	Organic Matrix: AUDMA, AFM, diurethane-DMA, 1,12-dodecane-DMA Filler: nonagglomerated/nonaggregated silica particles, zirconia particles, aggregated zirconia/silica clusters, and ytterbium trifluoride agglomerate particles 76.5 wt%	Increments of 2 mm.	2234800745	3M Oral Care, St Paul, MN, USA
Filtek Universal Capsule 0.2 g	Organic Matrix: AUDMA, AFM, diurethane-DMA, 1,12-dodecane-DMA Filler: nonagglomerated/nonaggregated silica particles, zirconia particles, aggregated zirconia/silica clusters, and ytterbium trifluoride agglomerate particles 76.5 wt%	Increments of 2 mm (injection). Heating to 70 °C is recommended by the manufacturer.	9934700	3M Oral Care, St Paul, MN, USA
Filtek One Bulk-Fill Capsule 0.2 g	Organic Matrix: AUDMA, diurethane- DMA, 1,12-dodecane- DMA Filler: Individualized and aggregated silica and zirconia, ytterbium trifluoride 76.5 wt%	Increments of 5 mm (injection).	2326500463	3M Oral Care, St Paul, MN, USA
Admira Fusion X-tra Bulk-Fill Capsule 0.2 g	Organic Matrix: Ormocer matrix Filler: barium aluminum borosilicate glass, pyrogenic silicon dioxide 84.0 wt%	Increments of 4 mm (injection).	2307439 2310563	VOCO, Cuxhaven, Germany
VisCalor Bulk-Fill Capsule 0.25 g	Organic Matrix: Bis-GMA, aliphatic dimethacrylate Filler: silicon dioxide 83.0 wt%	Increments of 4 mm (injection). Heating to 68 °C is recommended by the manufacturer.	2247155 2327944	VOCO, Cuxhaven, Germany
Ultra-Ecth Phosphoric acid at 35%	35% phosphoric acid, water, cobalt aluminate blue spinel, glycol, siloxane	Etchant.	BS8F6	Ultradent, Indaiatuba, SP, Brazil
Single Bond Universal Plus	10-MDP, HEMA, dimethacrylate resins, silanes (APTES/y-MPTES), silica, ethanol, water, and CQ	Adhesive.	9661952	3 M Oral Care, St Paul, MN, USA

All the materials’ data were provided by the manufacturers. AUDMA: aromatic urethane dimethacrylate; AFM: addition of fragmentation monomer; DMA: dimethacrylate; Bis-GMA: bisphenol-A glycol dimethacrylate; 10-MDP: methacryloxydecyl dihydrogen phosphate; HEMA: 2-hydroxyethyl methacrylate; APTES: 3-(aminopropyl)triethoxysilane; MPTES: y-methacryloxypropyltriethoxysilane; CQ: camphorquinone.

**Table 3:** Percentage (%) of internal voids.

Resin Composite	Room Temperature (24 °C) Mean and Standard Deviation (%)	Rank (24 °C)	Preheated (68 °C) Mean and Standard Deviation (%)	Rank (68 °C)
Filtek Universal	1.89 ^Aa^ ± 1.50	72.6	0.26% ^Ab^ ± 0.39	46.4
Filtek One Bulk-Fill	0.07 ^Ba^ ± 0.14	27.3	0.15% ^Aa^ ± 0.24	42.6
Admira Fusion X-tra Bulk-Fill	0.29 ^Aa^ ± 0.29	56.7	0.03% ^ABb^ ± 0.04	26.4
VisCalor Bulk-Fill	0.07 ^Ba^ ± 0.10	32.3	0.02% ^Ba^ ± 0.02	20.9

Uppercase letters in the columns and lowercase letters in the rows differ statistically according to the Tukey test performed on the ranks.

**Table 4:** Percentage (%) of continuous margins in enamel.

Resin Composite	Room Temperature (24 °C) Mean and Standard Deviation (%)	Rank (24 °C)	Preheated (68 °C) Mean and Standard Deviation (%)	Rank (68 °C)
Filtek Universal	86.4 ^B^ ±8.5	18.8	90.8% ^B^ ± 10.4	31.7
Filtek One Bulk-Fill	93.7 ^AB^ ± 8.1	35.6	94.8% ^AB^ ± 8.4	42.0
Admira Fusion X-tra Bulk-Fill	96.2 ^A^ ± 4.9	42.9	96.1% ^A^ ± 8.5	46.7
VisCalor Bulk-Fill	100 ^A^ ± 0.0	56.0	99.0% ^A^ ± 2.8	52.8

The different letters in the columns differ statistically according to the Tukey test performed on the ranks.

**Table 5:** Percentage (%) of continuous margins in dentin.

Resin Composite	Room Temperature (24 °C) Mean and Standard Deviation (%)	Rank	Preheated (68 °C) Mean and Standard Deviation (%)	Rank
Filtek Universal	75.1 ^C^ ± 20.6	19.90	77.9 ^C^ ± 18.1	25.10
Filtek One Bulk-Fill	87.2 ^BC^ ± 9.2	33.10	87.3 ^BC^ ± 10.3	34.30
Admira Fusion X-tra Bulk-Fill	89.8 ^B^ ± 6.2	36.20	95.7 ^AB^ ± 7.1	53.40
VisCalor Bulk-Fill	98.3 ^A^ ± 5.3	60.40	99.4 ^A^ ± 1.8	63.20

The different letters in the columns differ statistically according to the Tukey test performed on the ranks.

## Data Availability

The original contributions presented in this study are included in this article, and further inquiries can be directed to the corresponding author.
